# Sexual violence and associated factors among female large-scale industry workers in Bahir Dar city administration, Amhara region, Ethiopia

**DOI:** 10.11604/pamj.2024.47.213.36732

**Published:** 2024-04-29

**Authors:** Melkamu Atalel, Eleni Admassu, Zemenu Shiferaw Yadita

**Affiliations:** 1 Gozamen District Health Office, East Gojjam Zonal Health Department, Amhara Regional Health Bureau, Bahir Dar, Ethiopia; 2 Department of Reproductive Health and Population Studies, School of Public Health, College of Medicine and Health Science, Bahir Dar University, Bahir Dar, Ethiopia

**Keywords:** Associated factors, Bahir Dar, Ethiopia, large-scale industry, sexual violence

## Abstract

**Introduction:**

sexual violence is currently a serious public health problem affecting women´s health. Globally, 1 in 3 women faces sexual violence in their lifetime. Female industry workers are at an increased risk of sexual violence. Assessing the magnitude and factors associated with sexual violence among female industrial workers is important for interventions. The objective was to assess the prevalence and factors associated with sexual violence among female large-scale industries workers in Bahir Dar, Ethiopia, 2021.

**Methods:**

institution-based cross-sectional study was conducted on 807 female industry workers from September to October 2021. Participants were selected by systematic random sampling. The data were collected by a structured questionnaire. Data entry and analysis were done by Epi data v.3.1 and SPSS v.23, respectively. Multivariable logistic regression analysis was done to identify factors. Adjusted odds ratios were computed at 95%CI. A P-value below 0.05 was used to declare association.

**Results:**

the prevalence of sexual violence were 59.4% (95% CI; 56.0%-62.6%). The significantly associated factors include; age less than twenty-five (AOR=4.01, 95%CI; 2.81, 10.83), never-married women (AOR=3.07, 95%CI; 1.11, 8.46), being secondary education (AOR=2.65, 95%CI; 1.51, 4.66), being contract employee (AOR=4.65, 95%CI; 1.92, 11.22), drinking alcohol (AOR=3.01, 95%CI; 1.49, 6.09), and night work shift (AOR=9.01, 95%CI; 4.53, 17.93).

**Conclusion:**

high rate (59.4%) of sexual violence was reported. Age, marital status, educational status, contract type of work agreement, drinking alcohol, and working night work shift were risk factors. Hence, emphasis on creating safe working environment & transportation, education on reproductive rights and reporting of sexual violence.

## Introduction

According to the World Health Organization (WHO) definition, sexual violence is any sexual act, a sexual act attempt, sexual comments which are unwanted or an act of traffic directed against a person´s sexuality using force, by any person regardless of their relationship to the victim, and in any setting, such as home and work [1]. It can include the following; the threat of attempted rape, completed rape, sexual harassment, and sexual contact with the force. Sexual violence is a serious public health and human rights problem with both short and long-term consequences on women´s physical mental and sexual health [2]. It is one of the main contributors to poor sexual and reproductive health among women, leading to unintended pregnancy, self-induced abortions, gynecological problems, sexual dysfunction, and sexually transmitted infections (STIs), including HIV [3]. Worldwide, 35% of women on average have experienced sexual violence by someone who is a partner or not a partner [4]. It is a common problem to see sexual violence in low and middle-income countries, and the prevalence of sexual violence against women (VAW) is between 6% and 59% [5]. The rate of violence against women in African countries is 36.3% [6]. Similarly, in sub-Saharan African countries the rate of sexual violence is high i.e. 59% of ever-partnered women experienced sexual violence [7]. In Ethiopia, sexual violence against women is a common phenomenon. Nowadays, its magnitude is alarming not only in rural areas of the country but also in urban areas [8]. Ethiopia has a prevalence of sexual violence about 33% of women in a survey reported that they were physically forced to have intercourse [9].

Evidence showed that sexual violence and harassment unreasonably affect women, and men tend to be the perpetrators [10]. Unlawful Harassment and sexual violence are reported at an alarming rate among low-paid workers in the workplace [11]. Vulnerability to coerced sex and an increasing rate of HIV/AIDS infection are common among young women and girls [10]. Night shift work, alcohol intake, and working with third parties such as clients, customers, or users are risk factors for violence and harassment against women [12]. There is an increased risk of various forms of sexual violence among industry workers [13]. The workers are more vulnerable to abuse by employers or anyone from a co-worker due to the location of the work site and work characteristics [14]. The vulnerability of female industry workers is the undistinguishable site of work [15]. Sexual violence is a pervasive problem, but little is known about the prevalence and associated factors of sexual violence among females in the industry. In the developing world, there is a scarcity of data on sexual violence against female industry workers; and it is difficult to accurately estimate the prevalence of sexual violence. In general, sexual violence is a widely underreported public health issue, thus available data tend to underestimate the true scale of the problem. In addition, it is also a neglected area of research, and understanding the issue is crucial in order to promote a coordinated movement against it [16]. In Ethiopia, particularly in the Amhara region, there is a dearth of evidence on the magnitude and risk factors of sexual violence towards female large-scale industry workers. Hence, this study aimed to assess the prevalence of sexual violence and associated factors among large-scale industry workers. The findings of this study give programmatic and policy direction to tackle sexual violence among female industry workers in Ethiopia.

## Methods

**Study design**: an institution based cross-sectional study was conducted to determine the prevalence and associated factors of sexual violence among female large-scale industry workers at Bahir Dar city from September to October 2021.

**Setting and period**: the study was conducted at Bahir Dar city administration, which is the capital city of Amhara regional state, North-west Ethiopia. According to unpublished data of the Amhara regional state industry and investment bureau, there were 210 industries by 2020/2021 in the city. Among these, 67 of them are labeled as “large-scale industries” as per the criteria of the industry and investment bureau.

**Population**: the source populations were all female workers who were employees of large-scale industries in Bahir Dar city. The study populations were all female workers who were employee of randomly selected large-scale industries in Bahir Dar city.

**Inclusion criteria**: all-female workers who were working in the selected large-scale industries of Bahir Dar city were included in the study.

**Exclusion criteria**: those female workers who were unable to respond (e.g., who were unable to talk) or were very sick were excluded.

**Sample size**: the sample size was calculated by using a single population proportion formula. The assumptions were 50% prevalence of sexual violence among female large-scale industry workers, 5% margin of error, 95% confidence interval (CI), design effect of 2, and 5% non-response rate. The final sample size was 807.

**Sampling procedure**: cluster random sampling techniques were employed to select the study participants. Initially, sixty-seven (67) large-scale industries were identified in the Bahir Dar city administration, and each of them was considered a cluster. Then, the lottery method was used to select 14 (20%) of the large-scale industries. The illegible numbers of female workers were taken from the selected large-scale industries. Then, the total sample size was proportionally allocated to each selected large-scale industry. Finally, a systematic random sampling technique was employed to select each study participant.

**Variables**: the dependent variable was sexual violence. The independent variables includes; Socio demographic variables: Age, Religion, Educational level, Marital status, Residence (grow up), Work experience, Length living in the town, Income; Life style factors of employee: Alcohol, Chat chewing; Sexual History: Having history of sex, Age at initiation of sex, History of forced sex, multiple sexual partners, History of Rape or attempt of rape; Family history: Educational status of family; and Work characteristics: type of work, type of industry, working at night, work agreement.

**Data collection tools**: data were collected by using a structured questionnaire through face-to-face interviews. The questionnaire was adapted from WHO and other literature [1,17,18]. It contains socio-demographic characteristics, family history, sexual behaviors, work characteristics, and substance and alcohol use. The questionnaire was prepared in English and was translated to Amharic (local language) for appropriateness and easiness. The Amharic (local) versions were again retranslated back to English to check for consistency of meaning. The study participants were also informed about the confidentiality of the information and no personal identifiers were collected. Finally, filled questionnaires were signed by the principal investigator after checking for their completeness. To ensure data quality, training was given to all the data collectors and supervisors. In addition, the questionnaire was pre-tested on 10% of the sample size in Debre Markos town. The collected data were reviewed and checked for completeness before data entry.

### Measurement

***Sexual violence***: when a female large-scale industry worker experienced one of the following: being forced to have sexual intercourse against her will, having sexual intercourse due to fear of someone´s action, being forced to do something sexual that she thought was humiliating [16].

***Attempted rape***: the attempt to have non-consensual sexual intercourse with female large-scale industry worker whereby she was having a chance of escaping the attempt [19].

***Completed rape***: physically forced or otherwise coerced penetration of the vulva or anus using a penis [20].

***Life time rape***: when a female large-scale industry worker experienced rape in her life time [20].

***Sexual harassment***: when the participant experienced one of the following: unwanted and repeated sexual advances that range from unwelcome comments, kissing and touching in working environment” [21].

***Large scale industries***: are referred to as those industries that are having huge infrastructure, raw material, high manpower requirements and large capital requirements [22].

***Alcohol drinking***: female workers drink alcohol at least ones a week prior to the data collection [23].

**Data processing, analysis and interpretation:** all the questionnaires were coded and entered using Epi-data 3.1. Then data were exported to SPSS version 23 and cleaned to check for completeness. Descriptive analysis was done and presented with tables, graphs, frequencies, and texts. Binary logistic regression was also employed, the first bivariate logistic regression analysis was done to see the association of each independent variable with the outcome variable, and crude odds ratio (COR) with 95% CI was obtained. To improve the predictive ability of the factors, only variables having a p-value less than 0.25 were entered into the multivariable logistic regression. In the multivariable logistic regression, the association of each independent variable with the outcome variable was checked and possible confounding effects were controlled. Those variables with a p-value less than 0.05 in the multivariable logistic regression model were considered statistically significant. The adjusted odds ratio (AOR) was calculated at 95% CI as a measure of association. Multi-collinearity between each predictor was checked by a variance inflation factor (VIF). Hosmer-Lemeshow goodness of fit test was assessed for the goodness of the regression model.

**Ethical approval and consent to participate:** a letter of ethical approval was taken from the Bahir Dar University Research Ethics Review Board (Protocol. No. 275/2021). A letter of permission was also obtained from the Bahir Dar city administration industry and investment office and other concerned bodies to conduct the study. Written informed consent was obtained and respondents have been notified of the right to refuse or terminate at any point of the interview. For those participants who were unable to read the consent, a verbal explanation was given to them and their consent was recorded by the data collectors. The data given by the participants was used only for research purposes and kept confidential. Individual identifiers were removed to maintain the anonymity of respondents by assigning a unique number to each questionnaire. All data were entered into a password-protected computer.

## Results

**Socio-demographic characteristics**: a total of 807 female large-scale industry workers in Bahir Dar city have participated in this study, giving a 100% response rate. Of the total participants, 166 (20.6%) of them were below 25 years old. The mean age was 30 years with a Standard deviation of 6.5. The majority 733 (90.8%) of respondents were Orthodox Christians, and a monthly 274 (34%) of workers reported that they were never married, 386 (47.8%) of them attained a degree and above. Above a quarter of the respondents (29.9%) have a work experience of ten years and above. The majority (89.1%) of respondents were permanent employees of the industries. Only 10.2% of female workers think that their salary was enough for their monthly expenditure (Table 1).

**Table 1:** socio-demographic characteristics and family history of female large-scale industry workers in Bahir Dar city, North-west Ethiopia, 2021

Variables	Response	Number	Percent
**Age**	<25 years	166	20.6
	26-35 years	531	65.8
	>35 years	110	13.6
**Religion**	Orthodox	733	90.8
	Muslim	59	7.3
	Protestant	13	1.6
	Catholic	2	0.2
**Marital Status**	Never married	274	34.0
	Married	429	53.2
	Divorced	54	6.7
	Widowed	50	6.2
**Educational level of participants**	Grade 9-12	136	16.9
	Certificate/diploma	285	35.3
	Degree and above	386	47.8
**Monthly income of participants in Birra**	<1999	101	12.5
	2000-3999	245	30.4
	>4000	461	57.1
**Place of grow up**	Rural	317	39.3
	Urban	490	60.7
**Work experience**	<4 years	210	26.0
	5-9 years	356	44.1
	>10 years	241	29.9
**Type agreement of employment with this industry**	Contract	88	10.9
	Permanent	719	89.1
**Currently live with**	Alone	142	17.6
	With my family	236	29.2
	With husband/boyfriend	429	53.2
**Family condition**	Father and mother alive	520	64.4
	Only father alive	156	19.3
	Only mother alive	68	8.4
	Father and mother not alive	63	7.8
**Father and mother alive and live together now**	Yes	446	85.8
	No	74	14.2
**Receiving enough money based on demand**	Yes	82	10.2
	No	725	89.8

NB: a1 USD were exchanged 39.44 birr in 2021

**Work characteristics, and sexual history of participants**: the majority (80.4%) of the female workers were production staff the majority also work on both day and night shifts. Four hundred twenty-two (65.1%) of the night shift workers reported working six hours and above at night. Greater than half (54%) of them work in a department having greater than 10 staff members. One hundred ninety-eight (24.5%) of them drink alcohol and 50 (6.2%) of them chew chat. The majority (93.3%) of study participants had a history of sexual intercourse. More than one-third (67.7%) of them started sexual intercourse at 20 years and below. Three hundred ninety (48.3 %) of the sexually active respondents reported that they have had more than one sexual partner in their lifetime. Five hundred twenty (64.4%) of the participants were sexually active during the last 12 months before the study (Table 2).

**Table 2:** work characteristics and sexual history of female large-scale industry workers in Bahir Dar city, North-west Ethiopia, 2021

Variables	Response	Number	Percent
**Type of factory now working**	Garment	177	21.9
	Bottling	189	23.4
	Food processing	142	17.6
	Manufacturing	299	37.1
**Type of work**	Production staff	649	80.4
	Administrative staff	158	19.6
**Work shift**	Day	158	19.6
	Both day and night	649	80.4
**Length of working hours at night**	Less than six-hour	227	34.9
	Six hour and above	422	65.1
**Number of peoples work in the department**	1-2	230	28.5
	3-10	141	17.5
	>10	436	54.0
**Drink alcohol**	Yes	198	24.5
	No	609	75.5
**Frequency of drinking**	Regularly	47	5.8
	Sometimes	151	18.7
**Chewing chat**	Yes	50	6.2
	No	757	93.8
**Frequency of chewing**	Regularly	14	1.7
	Sometimes	36	4.5
**Ever had sexual intercourse**	Yes	753	93.3
	No	54	6.7
**Age at first sexual intercourse**	< 20 year	546	67.7
	>20 year	207	25.7
**Reasons to start sexual intercourse**	In a marriage	190	23.5
	Personal desire	508	62.9
	Promising word from partner	55	6.8
**Sexual intercourse in the last 12 months**	Yes	520	64.4
	No	287	35.6
**Number of sexual partners ever experienced Until now**	Nothing	54	6.7
	One	363	44.98
	Two	85	10.5
	Three	168	20.8
	Four and more	137	17.0

**Prevalence of sexual violence and perpetrators characteristics**: the overall lifetime prevalence of sexual violence among females working in large-scale industries was 59.4% (95% CI: 56.0%, 62.6%). Unwanted sexual act such as verbal jokes to have sex was experienced by 57.1%, and unwelcomed touching on genitalia or breast was reported by 39.3% of the study participants. Nearly one-third (27.5%) of the study participants had attempted rape, while 24.9% of them had completed rape in their lifetime. However, 9.3% of the study participants had completed rape 12 months prior to this study. About half of the perpetrators were co-workers, and most sexual violence occurs in the workplace which was 46.77%. The mechanism used to force the victims into sex differs were, 45(22.4%) mentioned that it was threatened by work, and 42(20.9%) reported that it was after they were threatened with harm. The majority of the girls 103(51.2%) reported that the perpetrators were older than the victims. Regarding the place of sexual violence (rape) most sexual violence occurs in the workplace which was 46.77% in hotels 22.39% on the street 21.39% in friend´s homes 6.9% and in the jungle 2.4% (Table 3).

**Table 3:** prevalence of sexual violence and perpetrators characteristics among female large-scale industry workers in Bahir Dar city, North-west Ethiopia, 2021

Variables	Response	Number	Percent
**Life time completed rape**	Yes	201	24.9
	No	606	75.1
**Completed rape in the last 12 month**	Yes	75	9.3
	No	732	90.3
**Sexual harassment**			
Un wanted sexual act to have sex	Yes	461	57.1
	No	346	42.9
Un welcome kissing	Yes	283	35.1
	No	524	64.9
Un welcome touching on genitalia or breast	Yes	317	39.3
	No	490	60.7
**Attempted rape**	Yes	222	27.5
	No	585	72.5
**Overall sexual violence**	Yes	479	59.4
	No	328	40.6
**The perpetuator was**	Close relative	45	22.4
	Co-worker	100	49.8
	Unknown person	9	4.5
	By group	47	23.4
**The mechanism used to force**	Hit me	26	12.9
	Pointed Knife	88	43.8
	Threats of harm	42	20.9
	Threatening by work	45	22.4
**Age of the perpetuator**	Older than me	103	51.2
	Younger than me	34	16.9
	Same age	64	31.8
**Place where the act performed**	Work place	94	46.8
	On the street	43	21.4
	Hotel	45	22.4
	Friends Home	14	7.0
	In the jungle	5	2.5

**Violence disclosure avoidance and reaction to the event**: after the unwanted sexual intercourse, 40.8% of them doesn´t share it with anyone and 87.2% didn´t report the event to legal bodies because do not know what to do (17.2%), they were afraid of their parents (21.2%), they were afraid the public reaction (18.2%), afraid of the Perpetuator (31.4%), the legal body is not helpful (12.0%). Among the perpetuators, 5(4.2%) were punished by imprisonment (Table 4).

**Table 4:** violence disclosure avoidance and reaction to the event by female forced sex victims among female large-scale industry workers in Bahir Dar city, North-west Ethiopia, 2021

Variables	Response	Number	Percent
Mechanism to escape from the attempted rape	By giving an appointment	77	34.7
	By getting help from another person	54	24.3
	By running	91	41.0
Shared the event after un wanted sexual intercourse	Nobody	82	40.8
	Friend	18	8.9
	Sister/Brother	13	6.5
	Health professional	88	43.8
Report the event to the legal body after the event	Did not reported	175	87.2
	To police	14	6.9
	Women's affair	12	5.9
Reasons for refusing to disclose violence	Do not know what to do	30	17.2
	Afraid of parents	37	21.2
	Afraid the public Reaction	32	18.2
	Afraid of the Perpetuator	55	31.4
	The legal body is not helpful	21	12.0
Action taken to the perpetuator	Nothing	95	79.84
	Imprisonment	5	4.2
	Financial compensation levied by elderly	11	9.24
	Suspended from work	8	6.72

**Factors associated with sexual violence**: all variables associated with sexual violence in the bivariate analysis at a p-value below 0.25 were entered into the multivariable logistic regression model in order to control confounding factors. In the multivariable logistic regression; Age, marital status, educational status, type of work agreement, drinking alcohol, and working shift, were the factors found to be statistically associated with sexual violence with a p-value less than 0.05. Consequently age < 25 years (AOR=4.01, 95%CI; 2.81, 10.83), age between 26 and 35 years (AOR=2.84, 95% CI; 1.04, 6.23), never married women (AOR=3.07, 95% CI; 1.11, 8.46), being married (AOR=0.29, 95% CI; 0.10, 0.83), educational status of 9-12 (AOR=2.65, 95%CI; 1.51, 4.66), Being contract employee (AOR=4.65, 95%CI; 1.92, 11.22), drinking alcohol (AOR=3.01, 95%CI; 1.49, 6.09), and night shift work (AOR=9.01, 95%CI; 4.53, 17.93) were factors found independently associated with sexual violence at p-value of less than or equals to 0.05. The odds of experiencing sexual violence among females whose age is less or equal to 25 years old were four times (AOR=4.01, 95%CI; 2.81, 10.83), more likely than those who were greater or equal to 36 years old. The odds of experiencing sexual violence were nearly three times (AOR=3.07, 95% CI; 1.11, 8.46) likely among never-married when compared to divorced or widowed women. Those female workers who attained grades 9-12 were at more than two times (AOR=2.65, 95%CI; 1.51, 4.66) greater risk of sexual violence as compared to female workers who attend above grade 12. On the other hand, contract employees were four times (AOR=4.65, 95%CI; 1.92, 11.22), more likely to encounter sexual violence than permanently employed female workers. Female large-scale industry workers who drink alcohol experience sexual violence three times (AOR=3.01, 95%CI; 1.49, 6.09), more likely than those who don´t drink alcohol. The odd of sexual violence was nine times more (AOR=9.01, 95%CI; 4.53, 17.93) likely among night shift female workers than those who had no night shifts (Table 5).

**Table 5:** factors associated with sexual violence among female large-scale industry workers in Bahir Dar city, North-west Ethiopia, 2021

Variables	Response	Sexual violence		COR (95%CI)	AOR (95%CI)	p value
		Yes	No			
**Age**	< 25 years	124	42	3.96(2.37, 6.62)	4.01(2.81, 10.83)	<0.001
	26-35 years	308	223	1.85(1.22, 2.80)	2.84(1.04, 6.23)	0.001
	>35 years	47	63	1	1	
**Marital status**	Never married	253	21	8.83(4.89, 15.96)	3.07(1.11, 8.46)	<0.001
	Married	166	263	0.46(0.30, 0.72)	0.29(0.10, 0.83)	0.001
	Divorced/Widowed	60	44	1	1	
**Educational level**	9-12	98	38	2.37(1.55, 3.63)	2.65(1.51, 4.66)	0.001
	Certificate/diploma	180	105	1.58(1.15, 2.16)	1.53(0.94, 2.50)	0.088
	Degree and above	201	185	1	1	
**Monthly income (ETB)**	≤ 1999	86	15	6.12(3.43, 10.91)	3.388(1.191,9.64)	0.221
	2000-3999	170	75	2.42(1.74, 3.36)	1.566(0.940,2.60)	0.085
	≥ 4000	223	238	1		
**Work experience (Years)**	< 4	176	34	2.87(1.83, 4.51)	1.373(0.701,2.69)	0.335
	5-9	148	208	0.395(0.28, 0.55)	0.318(0.185,0.55)	0.023
	≥ 10	155	86	1	1	
**Type of agreement in industry**	Contract	57	31	1.29(0.81, 2.05)	4.65(1.92, 11.22)	0.001
	Permanent	422	297	1	1	
**Drinking alcohol (employee)**	Yes	114	84	1.10(0.79, 1.52)	2.95(1.42, 6.93)	0.001
	No	365	244	1	1	
**Night shift**	Yes	419	230	2.97(2.08,4.26)	9.01(4.53, 17.93)	<0.001
	No	60	98	1	1	

## Discussion

In this study, we found that about sixty percent of female large-scale industry workers in Bahir Dar city experienced at least one form of sexual violence. Age, marital status, educational status, contract type of work agreement, drinking alcohol, and working night work shift were factors found independently associated with sexual violence. The overall lifetime prevalence of sexual violence which is rape attempted rape and sexual harassment among females working in a large factory was 59.4% with a 95% CI of (56.0%, 62.6%). This result was higher as compared to a study conducted among female administrative staff of Mekelle University which were 34.3% [24]. The finding of this study is in line with the study in Iran which was that 56% of Iranian female workers experienced some kind of sexual violence in the workplace. In addition, a study done to assess sexual harassment in the workplace conducted in Lebanon, the prevalence of sexual violence was 41.9% [25]. Furthermore, the finding of this study was greater than a study done in Nepal that assessed sexual violence in the work place, which showed about 53.8% of women employees faced sexual violence in their workplace [26]. Similarly, a study done in Malaysia among employed women found that 52.7% of sexual violence was conducted in the workplace. This variation might be due to differences in study participants and sample size.

Age is one of the socio-demographic variables found to be independently associated with sexual violence in females working in large industries. Age less than twenty-five years old females were four times more likely to experience sexual violence than older ones. This finding is consistent with the study done in Bishoftu town showed that younger age females in the age group 15-25 years were at greater risk for sexual violence than elders [27]. Similar studies in the U.S showed that college females between 18-24 years old experienced higher rates of sexual violence compared to other age groups [28]. This might be due to the fact that the lifestyles and routine activities approach suggests that younger women´s socialization behaviors might increase their exposure to motivated offenders who consider them suitable targets for sexual violence. This study also found that those women who were never married were three times at higher risk of sexual violence. This finding is consistent with the findings of a study in Iran [29]. These findings seem to indicate that unmarried women are particularly vulnerable to being victimized by non-strangers.

In this study, the participants who had night work shifts were nine times at greater risk than those who had not night shifts. In a study done at Mekele University female administrative staff female participants who had night work shifts were two times at greater risk than those who had no night shifts [24]. The finding of this study is consistent with the finding in Korea [30]. The reason behind the increased incidence of risk of sexual violence during the night shift might be night time is more convenient for violent people. In the present study participants who had a history of consuming alcohol were three times at greater risk of sexual violence than their counterparts. Our study is consistent with studies in Turkey, which revealed that sexual violence among university female students was about three times more likely common in students who had used alcohol than non-alcohol users [31]. The reason can be drinking alcohol may also place women in settings where their chances of encountering a potential offender are greater [14]. Educational status was also found to have a statistically significant association with sexual violence, in females grade 12 or lower with their educational status were three times at greater risk of sexual violence as compared to their counterparts. This study also in line with a study conducted in Debre Tabor which showed women who had low educational status were to increase the experience of sexual violence [32]. A comparable finding was obtained from a study conducted in Eastern Sudan, showed that women less than secondary education were more likely to experience sexual violence [33]. This is because the low educational status of women had no power to protect the violence.

In this study women´s who had Contract types of work agreement were also more likely to be a target of sexual violence with odd of which is approximately five times higher than those who were a permanent agreement. The finding of this study is in line with the study done in university women in Enugu, South-East Nigeria Women with temporary appointment had higher odds to report work place violence compared to women with a permanent employment status [34]. This might be due to contract employment of female workers experience violence due to non-existence or in effective mechanisms to deal with protective mechanism and fear of retribution. The strength of this study high response rate. However, this study was not supplemented with qualitative study which would enable to explore additional factors associated with sexual violence. This study was focused on victimization; we can only speculate about the motivation for perpetration; but perpetrators´ motivations for sexual violence was not assessed.

## Conclusion

This study has found a high prevalence of sexual violence among female industry workers in Bahr Dar city administration, compared to other studies. The factors associated with sexual violence in this study were; age below and equal to twenty-five, being never married, educational status 9-12 grades, being a contract employee, drinking alcohol, and working the night work shift were factors found independent predictors of with sexual violence among female industry workers in Bahr Dar city administration. Hence, this study suggests that the industry owners and managers should create a safe working environment & transportation. education about reproductive rights and reporting of sexual violence. Further studies should be done on perpetrators. The local and national health programs should give emphasis on women's empowerment & education, awareness creation regarding reproductive rights, and promotion of social values like marriage. Finally, we recommend the researchers should directly examine perpetrators´ motivations for sexual violence.

### What is known about this topic

- *Sexual violence is one of the major public health problems in the developing countries*.

### What this study adds

- *Significantly proportion of large industry female workers have experienced sexual violence;*
- *Being single increase sexual violence;*
- *Night shift female workers have increased risk of sexual violence*.
